# Die Rolle der körperlichen Aktivität in der Adipositasprävention

**DOI:** 10.1007/s00103-026-04310-x

**Published:** 2026-10-15

**Authors:** Christine Joisten, Nina Ferrari

**Affiliations:** Institut für Bewegungs- und Neurowissenschaft, Abteilung Bewegungs- und Gesundheitsförderung, Deutsche Sporthochschule Köln, Am Sportpark Müngersdorf 6, 50933 Köln, Deutschland

**Keywords:** Adipositas, Empfehlungen, Körperliche Aktivität, Sitzendes Verhalten, Lebensspanne, Obesity, Recommendations, Physical activity, Sedentary behaviour, Lifespan

## Abstract

Adipositas ist eine chronische, multifaktoriell bedingte Erkrankung, deren Entstehung durch genetische, neuroendokrine, psychosoziale und umweltbezogene Faktoren sowie durch Bewegungsmangel mitbestimmt wird. Körperliche Aktivität ist daher eine zentrale Säule der Prävention und Therapie. Neben dem Energieverbrauch beeinflusst sie Muskelmasse, Insulinsensitivität, Fettstoffwechsel, Entzündungsprozesse, psychische Gesundheit, Schlaf und funktionelle Kapazität. Dieser narrative Übersichtsbeitrag fasst aktuelle Leitlinien, Metaanalysen und repräsentative Monitoringdaten zur Rolle der körperlichen Aktivität über die Lebensspanne unter Berücksichtigung von Prävention und Therapie zusammen.

In der Prävention gelten zunächst die allgemeinen, altersspezifischen Empfehlungen für moderate bzw. intensive Bewegung sowie die Reduktion langer Sitzzeiten. In der Therapie ist der allein durch Bewegung erzielbare Gewichtsverlust bei Erwachsenen meist moderat; körperliche Aktivität ist jedoch unabhängig davon mit relevanten Verbesserungen von Körperzusammensetzung, Fitness, metabolischer Gesundheit, Mobilität und Lebensqualität assoziiert.

Aus Public-Health-Perspektive erfordert Bewegungsförderung zugleich verhältnisorientierte Ansätze, etwa durch sichere Fuß- und Radwege, attraktive und zugängliche Bewegungsräume, bewegungsfreundliche Bildungs- und Arbeitswelten sowie eine stigmavermeidende Versorgung. Physical Literacy als kompetenzbasiertes Konstrukt aus Motivation, Selbstvertrauen, körperlicher Kompetenz, Wissen und Verständnis kann dazu beitragen, körperliche Aktivität als langfristige Ressource für Gesundheit und Wohlbefinden und nicht allein als Mittel zur Prävention einer Gewichtszunahme oder zur Gewichtsreduktion zu verankern.

## Abstract (en)

Obesity is a chronic, multifactorial disease whose development is influenced by genetic, neuroendocrine, psychosocial and environmental factors as well as physical inactivity. Therefore, physical activity is a central pillar in the prevention and treatment of obesity. As well as increasing energy expenditure, it affects muscle mass, insulin sensitivity, fat metabolism, inflammatory processes, mental health, sleep and functional capacity. This narrative review summarises current guidelines, meta-analyses and representative monitoring data on the role of physical activity across the lifespan with regard to both prevention and treatment.

In terms of prevention, general and age-specific physical activity recommendations for moderate and vigorous activity and the reduction of prolonged periods of sitting apply. In terms of treatment, weight loss achievable through exercise alone is usually moderate in adults. However, physical activity is independently associated with significant improvements in body composition, fitness, metabolic health, mobility and quality of life.

From a public health perspective, promoting physical activity also requires environmental approaches, such as safe walking and biking paths, attractive and accessible spaces for physical activity, activity-friendly educational and work environments, and healthcare services that avoid stigmatization. Physical literacy—as a competency-based construct comprising motivation, self-confidence, physical competence, knowledge and understanding—can help to establish physical activity as a long-term resource for health and well-being, rather than merely as a means of preventing weight gain or losing weight.

## Einleitung

Der gesundheitliche Nutzen von körperlicher Aktivität ist über alle Altersgruppen hinweg umfassend belegt [1]. So ist Bewegung bei Kindern und Jugendlichen unter anderem mit einer besseren körperlichen Fitness, kardiometabolischen Gesundheit, Knochengesundheit, kognitiven Leistungsfähigkeit und psychischen Gesundheit sowie einem geringeren Körperfettanteil verbunden [2]. Analog weisen körperlich aktive Erwachsene nicht nur ein geringeres Risiko für Adipositas, sondern auch für Bluthochdruck, Diabetes mellitus Typ 2, ausgewählte Krebserkrankungen, Stürze und Osteoporose sowie eine geringere kardiovaskuläre und Gesamtsterblichkeit auf. Gleichzeitig sind damit eine bessere psychische und mentale Gesundheit, kognitive Leistungsfähigkeit und Schlafqualität assoziiert [1]. Definiert wird körperliche Aktivität als jede von den Skelettmuskeln erzeugte Körperbewegung, die zu einem Energieverbrauch führt [3]. Sport oder Sporttraining sind spezifische Formen der Bewegung, die geplant, strukturiert, repetitiv und zielgerichtet sind mit dem Ziel der Verbesserung oder Aufrechterhaltung einer oder mehrerer Komponenten der körperlichen Fitness.

Aufgrund des gesundheitlichen Nutzens empfiehlt die Weltgesundheitsorganisation (WHO) grundsätzlich: je mehr Bewegung, desto besser [1]. Allerdings unterscheiden sich die allgemeinen Bewegungsempfehlungen je nach Lebensalter und Lebensphase. Auf Basis der aktuell noch gültigen nationalen Bewegungsempfehlungen gilt für Säuglinge, dass sie sich so viel wie möglich bewegen sollten und ihr natürlicher Bewegungsdrang möglichst wenig eingeschränkt werden sollte [4]. Für noch nicht mobile Säuglinge schlägt die WHO zudem mindestens 30 min Bauchlage („tummy time“) über den Tag verteilt vor [2]. Für Kleinkinder und Kindergartenkinder werden mindestens 180 min körperliche Aktivität pro Tag empfohlen, verteilt über den Tag und in unterschiedlichen Formen und Intensitäten. Ab dem Kindergartenalter sollte davon ein Teil bereits in moderater bis intensiver Intensität erfolgen. Für Kinder ab 5 Jahren und Jugendliche empfehlen die WHO-Leitlinien durchschnittlich mindestens 60 min moderate bis intensive, überwiegend aerobe körperliche Aktivität pro Tag. Ergänzend sollen an mindestens 3 Tagen pro Woche intensive sowie muskel- und knochenstärkende Aktivitäten durchgeführt werden [2]. Die nationalen Empfehlungen liegen mit mindestens 90 min Bewegung pro Tag für Grundschulkinder und Jugendliche etwas höher, wobei ein Teil davon durch Alltagsaktivität erreicht werden kann. Zusätzlich wird auch hier eine höherintensive Beanspruchung großer Muskelgruppen an mehreren Tagen pro Woche empfohlen [5].

Für Erwachsene gelten 150–300 min moderate aerobe Aktivität pro Woche oder 75–150 min intensive Aktivität beziehungsweise eine Kombination aus beidem als Zielgröße. Ergänzend sollten an mindestens 2 Tagen pro Woche muskelkräftigende Aktivitäten durchgeführt werden. Bei älteren Erwachsenen entsprechen die Empfehlungen weitgehend denen für Erwachsene, werden jedoch um Gleichgewichts- und funktionelles Krafttraining ergänzt, insbesondere zur Sturzprävention.

In allen Altersgruppen wird außerdem betont, lange Sitzzeiten möglichst zu vermeiden beziehungsweise regelmäßig zu unterbrechen [6, 7]. *Sedentäres Verhalten* bezeichnet wach verbrachte Tätigkeiten mit sehr niedrigem Energieverbrauch von ≤ 1,5 metabolischen Äquivalenten (METs [metabolic equivalents of task]) in sitzender, zurückgelehnter oder liegender Körperposition [8]. *Körperliche Inaktivität* beschreibt dagegen das Nichterreichen alters- und lebensphasenspezifischer Bewegungsempfehlungen. In allen Altersklassen steigt mit langen und häufig wiederholten Sitzphasen das Risiko für nichtübertragbare Erkrankungen – zu denen auch die Adipositas selbst zählt – sowie für kardiometabolische Folgeerkrankungen [1].

Der vorliegende narrative Übersichtartikel basiert auf aktuellen deutsch- und englischsprachigen Leitlinien, Konsensuspapieren, systematischen Reviews und ausgewählten Primärstudien sowie auf zentralen Quellen der WHO, der Arbeitsgemeinschaft der Wissenschaftlichen Medizinischen Fachgesellschaften (AWMF), des Robert Koch-Instituts (RKI) und nationalen bzw. internationalen Fachgesellschaften. Der Schwerpunkt der integrierten Literatur lag auf den letzten 10 Jahren, allerdings wurden auch ältere Arbeiten, Definitionen bzw. grundlegende Studien berücksichtigt, wenn sie für Begriffe bzw. Konzepte maßgeblich waren.

Ziel dieses Beitrags ist, die Rolle der körperlichen Aktivität in der Prävention bzw. Therapie der Adipositas in allen Altersgruppen zu beleuchten. Zunächst werden Definition, Prävalenz, Ätiologie, Bewegungsempfehlungen und die Public-Health-Relevanz eingeordnet. Parallel soll auch die besondere Rolle der Reduktion von sitzendem Verhalten diskutiert werden, da auch damit gesundheitliche Vorteile unabhängig vom Ausmaß der regelmäßigen körperlichen Aktivität verbunden sind [9].

## Prävalenz und Ätiologie der Adipositas in Deutschland

Adipositas gilt heutzutage als chronische, multifaktoriell bedingte bzw. nichtübertragbare Erkrankung, die bei Erwachsenen meist über einen Body-Mass-Index (BMI) ≥ 30 kg/m^2^ definiert wird, während bei Kindern und Jugendlichen alters- und geschlechtsspezifische Referenzwerte herangezogen werden [7, 10]. Rubino et al. [11] schlugen 2025 vor, dass eine Adipositas nicht allein über den BMI, sondern als Zustand überschüssiger Fettmasse mit oder ohne abnorme Verteilung beziehungsweise Funktion verstanden werden soll. Diese Definition wird international noch diskutiert und ist bislang nicht in alle Leitlinien und in die klinische Routineversorgung übernommen; ihr Stellenwert für Diagnostik und Versorgung bleibt abzuwarten. Bemerkenswert ist aber die Unterscheidung zwischen einer präklinischen und klinischen Adipositas, Letztere geht mit Funktionsstörungen von Organen und Geweben einher [11].

Aktuell sind in Deutschland laut GEDA 2019/2020-EHIS 53,5 % der Erwachsenen von Übergewicht, einschließlich Adipositas, betroffen; die Adipositasprävalenz liegt bei Frauen und Männern jeweils bei 19,0 % [12]. Bei 3- bis 17-jährigen Kindern und Jugendlichen zeigten die Daten aus der zweiten Folgeerhebung der Studie zur Gesundheit von Kindern und Jugendlichen in Deutschland (KiGGS Welle 2, 2014–2017) eine Übergewichtsprävalenz von 15,4 % und eine Adipositasprävalenz von 5,9 % [13]. In beiden Altersbereichen bestehen ausgeprägte soziale Gradienten. So ist Adipositas in Gruppen mit niedriger Bildung beziehungsweise einem niedrigeren sozioökonomischen Status häufiger.

Die Adipositasentstehung als solche lässt sich nicht allein durch eine positive Energiebilanz erklären, da Energieaufnahme und -verbrauch, Hunger, Sättigung und Fettverteilung biologisch reguliert und zugleich sozial beeinflusst werden [14]. Genetische Faktoren beeinflussen unter anderem die Appetitregulation, Belohnungsverarbeitung und Energiehomöostase und damit die individuelle Vulnerabilität gegenüber adipogenen Umweltbedingungen [15]. Bewegung nimmt in der Entstehung, aber auch Bekämpfung eine Schlüsselrolle ein, weil sie nicht nur den Energieverbrauch erhöht, sondern auch die Muskelmasse, Insulinsensitivität, Fettstoffwechsel, Entzündungsprozesse, Schlafqualität, psychische Gesundheit und Appetitregulation beeinflusst [1, 16]. Besonders relevant ist dabei nicht nur Sport im engeren Sinn, sondern auch Alltagsbewegung, aktive Mobilität, Spiel, Treppensteigen, Gehen und Radfahren, da diese kumulativ zur Energiehomöostase beitragen. Umgekehrt begünstigen körperliche Inaktivität und langes Sitzen eine positive Energiebilanz, da sitzende Zeit den Energieverbrauch senkt und leichte Alltagsaktivitäten verdrängt.

Im Kindes- und Jugendalter wirken Bewegungsmangel, fehlende sichere Spiel- und Bewegungsräume, motorisierter Transport, Bildschirmzeit, Schulumgebung und Lebensmittelmarketing zusammen und können früh langfristige Risikopfade etablieren [2]. Im Erwachsenenalter verstärken beruflicher Sedentarismus, Schichtarbeit, Schlafmangel, Stress, Care-Arbeit (Betreuung und Pflege), psychische Erkrankungen und gewichtssteigernde Medikamente die Wirkung einer bewegungsarmen Lebensweise [1]. Im höheren Lebensalter wiederum erhält Bewegung eine zusätzliche Bedeutung, weil körperliche Inaktivität Muskelverlust, Funktionsabbau und sarkopene Adipositas (Adipositas bei gleichzeitig verminderter Muskelmasse und -funktion) begünstigen kann, während Kraft- und Ausdaueraktivität zum Erhalt von Muskelmasse und Mobilität beiträgt [17].

Insgesamt ist Adipositas daher als Ergebnis einer lebenslangen Wechselwirkung zwischen biologischer Vulnerabilität, früher Prägung, psychosozialen Belastungen, medizinischen Faktoren, strukturell obesogenen (adipositasfördernden) Ernährungsumwelten und bewegungsarmen Lebensbedingungen zu verstehen. Hinzu kommen soziale Determinanten wie Bildung, Einkommen, Wohnumfeld, Arbeitsbedingungen und Zugang zu Versorgung [18, 19].

## Status quo körperliche Aktivität in Deutschland und Lebensverlaufsperspektive

Repräsentative Daten aus der KiGGS Welle 2 zeigten, dass nur 22,4 % der Mädchen und 29,4 % der Jungen im Alter von 3 bis 17 Jahren täglich mindestens 60 min körperlich aktiv waren; die Erreichung der empfohlenen Umfänge nimmt mit dem Alter ab und niedrige Aktivitätsniveaus sind besonders häufig bei älteren Mädchen sowie bei Kindern und Jugendlichen aus Familien mit niedrigem sozioökonomischen Status zu beobachten [20, 21]. Daten der Studie „Health Behaviour in School-aged Children, HBSC“ aus 2022 bestätigen diese Geschlechterunterschiede für 11- bis 15-Jährige mit lediglich 10,8 % der Mädchen und 20,9 % der Jungen, die die tägliche Bewegungsempfehlung erreichten [22]. Neben dem Gewichtsstatus, Alter und Geschlecht beeinflussen sozioökonomische Lage, familiäre und Peer-Unterstützung, körperliches Selbstkonzept, Wohnumgebung und Zugang zu organisierten beziehungsweise unorganisierten Bewegungsangeboten das Bewegungsverhalten [23]. MoMo-(Motorik-Modul-) und KiGGS-Analysen zeigen, dass sozioökonomische Unterschiede besonders den Vereinssport und andere organisierte Sportformen betreffen, während unorganisierte Aktivität weniger stark sozial determiniert ist [23]. Weitere MoMo-Daten weisen zudem darauf hin, dass Kinder und Jugendliche mit Übergewicht seltener an organisierten Sportangeboten teilnehmen und bei unorganisierter Aktivität vor allem die Teilnahmehürde erhöht ist [24].

Im Erwachsenenalter erreichen in Deutschland nach dem WHO-Länderprofil 48 % der 18- bis 99-Jährigen die Ausdauerempfehlung, 36 % die Empfehlung zur Muskelkräftigung und 26 % beide Empfehlungen [25]. Gleichzeitig sind lange Sitzzeiten verbreitet, insbesondere bei Männern, jüngeren Erwachsenen und Personen mit höherer Bildung. In GEDA 2019/2020 berichteten 16,7 % der Frauen und 22,3 % der Männer mindestens 8 h Sitzzeit täglich; etwa ein Fünftel saß mindestens 4 h täglich und war zugleich in der Freizeit körperlich inaktiv [26].

Das Bewegungsverhalten zeigt über den Lebensverlauf nur eine niedrige Stabilität; aktive Kinder werden daher nicht automatisch aktive Erwachsene, wenngleich frühe positive Bewegungserfahrungen spätere Aktivität begünstigen können [27].

## Bewegung in der Individualprävention

Zur Adipositasprävention empfiehlt die aktuelle deutsche S3-Leitlinie für Erwachsene regelmäßige körperliche Aktivität im Alltag, in der Freizeit und im Beruf (Empfehlungsgrad A, Evidenzgrad moderat; [7]). Sie stützt sich unter anderem auf die systematische Übersichtsarbeit von Jakicic et al. [28]. Darin wurde gezeigt, dass höhere körperliche Aktivität mit einer geringeren Gewichtszunahme assoziiert ist bzw. ein dosisabhängiger Zusammenhang zwischen körperlicher Aktivität und der Prävention einer Gewichtszunahme besteht. Die berichteten Schwellenwerte lagen zwischen 60 min/Woche intensiver bzw. 150 min/Woche moderater körperlicher Aktivität und bis zu 300 min/Woche moderater Aktivität. Positive Effekte wurden für moderate und intensive, nicht jedoch für niedrigintensive körperliche Aktivität beobachtet. Auch Interventionsstudien stützen den Nutzen regelmäßiger körperlicher Aktivität zur Prävention einer Gewichtszunahme. So zeigte die Metaanalyse von Goryakin et al. [29], dass Interventionen zur Bewegungsförderung im hausärztlichen Setting den BMI geringfügig, aber statistisch signifikant reduzierten (−0,21 kg/m^2^).

Folgende möglichen Strategien zur Bewegungsförderung auf individueller Ebene wurden zusammengefasst [7]:

- Steigerung von Alltagsaktivitäten (wie z. B. Nutzung von Treppen statt Aufzügen oder Rolltreppen),
- Zurücklegen von Strecken zur Arbeit, zum Einkaufen, zu Bildungs- oder Freizeiteinrichtungen zu Fuß oder mit dem Fahrrad, bei längeren Strecken Kombination aus aktiven und inaktiven Fortbewegungsformen,
- aktive Gestaltung von Freizeit und Arbeitspausen in Abhängigkeit von persönlichen Vorlieben und Gegebenheiten, wie z. B. Spazierengehen, Fahrradfahren, Joggen, Schwimmen, Gymnastik, Wandern, Fitness, Ballsportarten, Gärtnern und Wintersport,
- Begrenzung der Bildschirmzeit, z. B. fernsehfreie Tage oder zeitliche Obergrenzen.

Für Kinder und Jugendliche:

- Altersangemessen spielerische und alltagsintegrierte Bewegung fördern
- Individuelle Interessen, Präferenzen und Motivation berücksichtigen
- Regelmäßige körperliche Aktivität in Kindergarten‑, Schul‑, Familien- und Freizeitalltag integrieren
- Regelmäßige Bewegungsmöglichkeiten in den Tagesablauf integrieren
- Aktive Wege im Alltag nutzen, z. B. zu Fuß gehen oder Fahrradfahren
- Längere Phasen sitzender Tätigkeiten vermeiden und bewegungsorientierte Alternativen zu Bildschirmzeit und Sitzen anbieten

## Bewegung in der Verhältnisprävention

In der aktuellen S3-Leitlinie zur Adipositasprävention wurden auch verhältnispräventive Maßnahmen adressiert. Dazu zählen sichere und attraktive Fuß- und Radwege, verkehrsberuhigte Quartiere, erreichbare Grün‑, Spiel- und Sportflächen, fahrrad- und fußgängerfreundlicher öffentlicher Verkehr, bewegungsfreundliche Schulen und Arbeitsplätze sowie barrierearme Angebote für Menschen mit eingeschränkter Mobilität [6, 7]. Die Evidenz zu verhältnispräventiven Bewegungsmaßnahmen ist allerdings je nach Setting, Zielgröße und Studiendesign heterogen. Ein 2015 veröffentlichtes Cochrane-Review untersuchte 33 Studien zu verhaltens- und verhältnispräventiven Maßnahmen zur Förderung körperlicher Aktivität auf Gemeindeebene. Die meisten Interventionen wurden von lokalen Behörden oder NGOs („non-governmental organizations“) umgesetzt (29 Studien) und umfassten überwiegend kommunikationsbasierte Strategien (26 Studien). Insgesamt zeigten die eingeschlossenen Studien jedoch nur begrenzte Evidenz für eine Wirksamkeit auf Bevölkerungsebene: In den meisten Untersuchungen konnten keine statistisch signifikanten Effekte auf das Bewegungsverhalten oder das Körpergewicht festgestellt werden.

Einzelne Studien berichteten jedoch über eine Zunahme der für Gehen oder Laufen aufgewendeten Zeit in bestimmten Bevölkerungsgruppen [30]. Verbesserungen der bebauten Umgebung sind konsistent mit höherer Alltagsaktivität und aktiver Mobilität assoziiert; direkte Effekte auf Adipositasmaße sind jedoch schwieriger nachzuweisen und häufig kontextabhängig. Arbeitsweltbezogene Maßnahmen wie Sitz-Steh-Arbeitsplätze können Sitzzeiten kurzfristig reduzieren, sollten aber möglichst mit aktiven Unterbrechungen, Gehpausen und organisatorischen Maßnahmen kombiniert werden. Aus Public-Health-Perspektive ist daher nicht die Einzelmaßnahme entscheidend, sondern ein mehrstufiger Ansatz, der individuelle Befähigung mit bewegungsfördernden Lebenswelten verbindet.

Zu den *Maßnahmen, die die Wahl bewegungsaktiverer Fortbewegungsmöglichkeiten fördern*, zählen u. a. (modifiziert zusammengefasst aus der S3-Leitlinie Adipositas [7]):

- Bau von ausreichend breiten, sicher und attraktiv gestalteten Fahrradwegen, Fußgängerzonen und Bürgersteigen,
- Einrichtung von sicheren, attraktiven Fahrradabstellvorrichtungen im öffentlichen Raum (z. B. an Bahnhöfen, S‑ und U‑Bahnstationen),
- Stärkung des öffentlichen Personennahverkehrs als zum Fahrrad- und Fußgängerverkehr komplementäres Angebot.

Zu den evidenzgestützten und kontextbezogen empfohlenen *Maßnahmen zur Förderung bewegungsaktiver Formen der Freizeitgestaltung* zählen unter anderem (modifiziert zusammengefasst nach S3-Leitlinie Adipositas [7]):

- Schaffung attraktiver Parkanlagen, Grün- und Naherholungsflächen mit Möglichkeiten bewegungsaktiver Freizeitgestaltung (wie z. B. Spazier- und Wanderwegen, Joggingstrecken, Trimm-dich-Pfaden),
- sichere und attraktive Spielplätze,
- Bau und ausreichende Finanzierung von Schwimmbädern, Sportplätzen und anderen öffentlichen Sportanlagen,
- Förderung von Sportvereinen und anderen Formen des Breitensports,
- Befreiung von Fitnessstudiobeiträgen und vergleichbaren Dienstleistungen von der Mehrwertsteuer.

Zu den *Maßnahmen zur Förderung eines bewegungsaktiven Arbeitsalltags* zählen u. a. die folgenden Maßnahmen:

- Bereitstellen von sicheren und attraktiven Fahrradabstelleinrichtungen,
- Bereitstellen von Duschen und Umkleiden,
- Bereitstellen von Stehpulten,
- Ermöglichung von aktiven Pausen durch flexible Arbeitszeiten,
- Einrichtung von Fitnessräumen,
- Förderung von Dienstfahrrädern und anderen bewegungsfreundlichen Alternativen zu Dienstwagen,
- bewegungsfreundliche Gestaltung von Gebäuden, u. a. die Ausschilderung von Treppen.

Zu den *Maßnahmen zur Förderung eines bewegungsaktiven Alltags für Kinder und Jugendliche* zählen:

- bewegungsförderliche Umgebungen im familiären Umfeld schaffen,
- körperliche Aktivität systematisch in Bildungseinrichtungen wie Kindergärten, Schule, Freizeit und kommunale Settings integrieren,
- niedrigschwellige Bewegungsangebote in bestehenden Strukturen wie Familie, Kita, Schule, Sportverein, Freizeitangeboten und kommunalen Programmen ermöglichen,
- schulische Bewegungsangebote und aktive Wege im Alltag strukturell unterstützen,
- alltagsintegrierte Bewegungsförderung priorisieren, weil sie praktikabel und langfristig leichter umsetzbar ist.

## Reduktion vermeidbarer Sitzzeiten

Inzwischen finden sich in nahezu allen Bewegungsempfehlungen konsequent Hinweise zur Reduktion vermeidbarer Sitzzeiten, etwa durch Stehpulte, Positionswechsel, kurze Steh- oder Gehpausen und eine aktive Gestaltung von Arbeits‑, Schul- und Alltagsroutinen. Die WHO-Leitlinie zu körperlicher Aktivität und sedentärem Verhalten gibt erstmals explizite Empfehlungen zur Reduktion von Sitzzeiten für Kinder, Jugendliche, Erwachsene, ältere Erwachsene sowie Schwangere und postpartale Frauen [1].

Der Einsatz von Sitz-Steh-Arbeitsplätzen kann den Energieumsatz gegenüber rein sitzender Schreibtischarbeit moderat erhöhen; Gibbs et al. fanden beim abwechselnden Sitzen und Stehen einen Anstieg des Energieumsatzes um 7,8 %, beim Stehen um 11,5 % [31]. Allerdings ist reines Stehen metabolisch weniger wirksam als kurze Gehpausen. So zeigten Creasy et al. [32], dass Gehen den Energieumsatz deutlich stärker steigerte als Sitzen oder Stehen. Daher empfahlen die Autoren, dass Sitzunterbrechungen möglichst als kurze Bewegungspausen und nicht nur als statisches Stehen umgesetzt werden sollten. Bei älteren Erwachsenen ist die Reduktion langer Sitzphasen besonders bedeutsam, weil lange Sitzzeiten mit ungünstigen metabolischen Veränderungen, insbesondere des Glukosestoffwechsels, sowie mit Adipositas, Funktionsverlust, Gebrechlichkeit (Frailty) verbunden sind und im Alltag zu eingeschränkter Mobilität und geringerer sozialer Teilhabe beitragen können [33]. Außerdem scheint die Sitzzeit bei älteren Erwachsenen positiv mit einer abdominalen Adipositas assoziiert zu sein; häufigere Unterbrechungen der Sitzzeit gehen dagegen mit günstigerer abdominaler Fettverteilung einher [34]. Für diese Altersgruppe sind daher niedrigschwellige Strategien besonders geeignet, wie beispielsweise wiederholtes Aufstehen vom Stuhl, kurze Gehpausen, leichte Haus- und Alltagsaktivität, Balanceübungen sowie sichere Kraft- und Mobilisationsübungen.

Bei Kindern und Jugendlichen sollten vermeidbare Sitzzeiten vor allem durch die Begrenzung übermäßiger Bildschirmzeit, aktive Schulwege, bewegungsfördernde Unterrichtselemente und spielerische Bewegungsangebote reduziert werden. Die WHO empfiehlt für 5‑ bis 17-Jährige, die insgesamt in sedentärem Verhalten verbrachte Zeit und insbesondere die freizeitbezogene Bildschirmzeit zu begrenzen; die vielfach genannte Obergrenze von etwa 2 h Bildschirmzeit pro Tag stammt aus nationalen bzw. pädiatrischen Empfehlungen und ist keine numerische WHO-Vorgabe [2]. Effekte auf den BMI oder Adipositas sind zwar klein beziehungsweise inkonsistent, daher wird eine Reduktion der Sitzzeit meist als Teil multimodaler Programme mit Bewegung, Schlaf, Ernährung und Familienunterstützung verstanden [35, 36].

## Alltagsaktivitäten

Als Alltagsaktivitäten werden Bewegungsformen und Tätigkeiten bezeichnet, die Menschen in ihrem täglichen Leben ausführen, eine leichte Intensität aufweisen und die keine spezifische sportliche Betätigung darstellen, zum Beispiel Gehen, Treppensteigen, Hausarbeit, Einkäufe, aktive Mobilität oder Gartenarbeit. Die WHO versteht körperliche Aktivität ausdrücklich nicht nur als Sport, sondern auch als Bewegung im Rahmen von Transport, Arbeit, Haushalt, Schule, Freizeit und Gemeinschaftsaktivitäten; entsprechend ist Alltagsbewegung gerade für Menschen mit Übergewicht oder Adipositas bedeutsam, weil sie niedrigschwellig, wiederholbar und langfristig in den Tagesablauf integrierbar ist [1]. Schrittzähler, Apps oder Wearables (z. B. Fitnessarmbänder) können diese Strategie unterstützen, indem sie Selbstmonitoring, Zielsetzung und Feedback erleichtern.

Heutzutage gelangen in diesem Kontext sogenannte Exercise Snacks zunehmend in den wissenschaftlichen Fokus. Darunter werden sehr kurze, über den Tag verteilte Bewegungseinheiten verstanden, etwa Treppensprints, kurze zügige Gehintervalle, Kniebeugen oder andere kurze intensive Bewegungsimpulse. Dieser Ansatz passt gut zur aktuellen WHO-Empfehlung, wonach jede körperliche Aktivität zählt und die frühere Mindestanforderung von mindestens 10 min pro Aktivitätseinheit aufgehoben wurde [1]. Jones et al. [37] beschrieben Exercise Snacks und andere Formen intermittierender körperlicher Aktivität als praktikabel und wahrscheinlich sicher für Erwachsene und ältere Erwachsene; die epidemiologischen Daten zeigten günstige Zusammenhänge kurzer intensiver Alltagsaktivität mit Mortalität und kardiovaskulären Endpunkten, während die Interventionsstudien vor allem moderate Effekte auf die kardiorespiratorische Fitness und noch uneinheitliche Effekte auf Funktion, Stimmung, Lebensqualität und weitere Gesundheitsendpunkte zeigten.

In eine aktuelle Metaanalyse schlossen Rodríguez et al. [38] 11 randomisierte Studien mit 414 körperlich inaktiven jüngeren und älteren Erwachsenen ein. Dabei wurden Exercise Snacks als strukturierte Belastungseinheiten von höchstens 5 mindefiniert, die mindestens 2‑mal täglich, an mindestens 3 Tagen pro Woche und über mindestens 2 Wochen durchgeführt wurden. Die Interventionen verbesserten signifikant die kardiorespiratorische Fitness und zeigten Verbesserungen der muskulären Ausdauer bei Älteren; keine signifikanten Effekte fanden sich jedoch auf Körperzusammensetzung, Blutdruck oder Blutfette. Besonders deutlich wurde aber die hohe Adhärenz mit mehr als 80 %.

Insgesamt ist die Evidenz zu Exercise Snacks jedoch noch heterogen und beruht überwiegend auf kleinen, kurzfristigen Studien; belastbare Aussagen zu Körpergewicht, Körperzusammensetzung und langfristigen Gesundheitseffekten sind daher derzeit nur eingeschränkt möglich. Für Kinder und Jugendliche können aktive Pausen die Konzentrationsfähigkeit steigern und gleichzeitig für mehr Ruhe und Wohlbefinden im Klassenraum sorgen [39].

## Bewegung als Therapiesäule

Während in der Prävention im Wesentlichen die allgemeinen Bewegungsempfehlungen gelten, liegen adipositasspezifische Bewegungsumfänge häufig oberhalb dieser Basisempfehlungen, insbesondere wenn nach einer Gewichtsreduktion eine langfristige Gewichtsstabilisierung erreicht werden soll (Tab. 1). Zwar stellen vermehrte körperliche Aktivität und die Reduktion vermeidbarer Sitzzeiten wichtige Säulen der Adipositastherapie dar [7]; der direkte Effekt von Bewegung allein auf die Gewichtsabnahme ist bei Erwachsenen jedoch meist moderat und liegt häufig nur im Bereich von etwa 2–3 kg. Dieser mittlere Effekt hängt allerdings stark von Trainingsumfang, Intensität, Ausgangsgewicht und Adhärenz ab; zudem sind Veränderungen der Körperzusammensetzung – insbesondere die Reduktion viszeraler Fettmasse und der Erhalt bzw. Aufbau fettfreier Masse – klinisch häufig bedeutsamer als die Veränderung des Körpergewichts allein.

**Tab. 1:** Körperliche Aktivität bei Adipositas nach Altersgruppen: Deutsche Leitlinien, internationale Empfehlungen und adipositasspezifische Einordnung

Zielgruppe	Deutsche Leitlinien (zusammengefasst aus [7])	Internationale Empfehlungen	Adipositasspezifische Quintessenz
Frühes Kindesalter (< 5 Jahre)	In der hier ausgewerteten deutschen Adipositas-Leitlinienlandschaft fand sich keine separat ausgewiesene aktuelle minutengenaue Adipositas-Empfehlung speziell für < 5-Jährige. Die zuletzt verfügbare deutsche S3 zu Adipositas im Kindes- und Jugendalter nennt 90 min moderate Aktivität/Tag für „Kinder und Jugendliche“ allgemein, ist aber nur bis 30.08.2024 gültig geführt; eine Aktualisierung ist angemeldet [48]	WHO: < 1 Jahr mehrmals täglich aktiv; bei noch nicht mobilen Säuglingen ≥ 30 min Bauchlage/Tag; 1–2 Jahre ≥ 180 min/Tag; 3–4 Jahre ≥ 180 min/Tag, davon ≥ 60 min moderat bis intensiv [49]	Im frühen Kindesalter stehen bei Adipositas eher familienbasierte, spielerische Aktivierung und die Reduktion von Inaktivität im Vordergrund als eine adipositasspezifische Trainingsdosis
Kinder und Jugendliche (5–17 Jahre)	Die letzte deutsche pädiatrische S3 [48] empfiehlt generell 90 min moderate Aktivität pro Tag, mit schrittweiser Steigerung bei bewegungsarmen Kindern/Jugendlichen. Das Training soll Kraft- und Ausdaueranteile enthalten, die Alltagsaktivität deutlich erhöhen, gelenkschonend sein, Inaktivität/Medienkonsum reduzieren, an den Grad der Adipositas angepasst werden und vom sozialen Umfeld unterstützt werden	WHO (5–17 Jahre): im Mittel ≥ 60 min/Tag moderat bis intensiv, überwiegend aerob, plus intensive sowie muskel- und knochenstärkende Aktivität an ≥ 3 Tagen/Woche [1]	Bei pädiatrischer Adipositas ist neben dem zeitlichen Umfang der Bewegung vor allem ein motivationsorientiertes, alltagsnahes und niedrigschwelliges Aktivitätskonzept von Bedeutung. Die deutsche Empfehlung ist mit 90 min Bewegung pro Tag dabei deutlich ambitionierter als die WHO-Empfehlung von 60 min/Tag
		NICE betont, dass Kinder/Jugendliche mit Übergewicht oder Adipositas mehr als die Standardempfehlung benötigen können; empfohlen werden sowohl Alltagsbewegung als auch regelmäßige strukturierte Aktivität, abgestimmt auf Interessen, Fähigkeit, Selbstvertrauen und Kosten [50]	
		Obesity Canada (gemeinnützige Fachorganisation) empfiehlt Bewegungsinterventionen als Teil der Adipositastherapie bei ≤ 18-Jährigen [51]	
Erwachsene (18–64 Jahre)	Die aktuelle deutsche S3 „Prävention und Therapie der Adipositas“ [7] empfiehlt zur Unterstützung der Gewichtsabnahme 30–60 min körperliche Aktivität pro Tag. Kontraindikationen sollen ausgeschlossen, Alltags- und Freizeitaktivität gesteigert und Betroffene auf gesundheitliche Vorteile unabhängig von Gewichtsverlust hingewiesen werden	WHO: ≥ 150–300 min/Woche moderat oder 75–150 min/Woche intensiv, plus Krafttraining ≥ 2 Tage/Woche; > 300 min/Woche bringen zusätzliche gesundheitliche Vorteile [1]	Erwachsene sind die am besten spezifizierte Gruppe. Die übereinstimmende Botschaft lautet: Für Gesundheit reichen oft die allgemeinen Basisziele; für Gewichtsreduktion und besonders für Gewichtserhalt nach Gewichtsverlust werden meist höhere Umfänge empfohlen
		EASO: für Reduktion der Fettmasse 150–200 min/Woche moderates Ausdauertraining; für Gewichtserhalt nach Gewichtsverlust 200–300 min/Woche; Krafttraining zum Erhalt der Magermasse; Hochintensitäts-Intervalltrainings (HIIT) nur nach kardiovaskulärer Risikoabschätzung und idealerweise supervidiert [40]	
		NICE: oft 45–60 min/Tag zur Prävention bzw. 60–90 min/Tag zur Vermeidung eines Weight Regain nach Gewichtsverlust [50]	
		Obesity Canada: 30–60 min moderat bis intensiv an den meisten Tagen, mit Nutzen für Viszeralfett, Fitness, Mobilität und Risikofaktoren auch ohne Gewichtsverlust [41]	
Ältere Erwachsene (≥ 65 Jahre)	Deutschland verweist auf dieselben Grundziele wie für jüngere Erwachsene; bei eingeschränkter Mobilität sollen zusätzlich Gleichgewichtsübungen an mindestens 3 Tagen/Woche erfolgen und bei Einschränkungen soll „so viel wie möglich“ körperlich aktiv geblieben werden. Die deutsche Erwachsenen-S3-Leitlinie betont zudem den Nutzen von Krafttraining zum Erhalt von Magermasse und Knochengesundheit bei älteren Menschen mit (sarkopener) Adipositas [7]	WHO: gleiche Basisziele wie Erwachsene, zusätzlich multikomponentige Aktivität mit Fokus auf funktionelles Gleichgewicht und Krafttraining an ≥ 3 Tagen/Woche [1]	Bei älteren Menschen mit Adipositas steht der Erhalt der körperlichen Funktionsfähigkeit im Vordergrund: Ziel ist nicht primär die Senkung des Body-Mass-Index (BMI), sondern die Vermeidung von Muskelabbau (Sarkopenie) und Gebrechlichkeit (Frailty), der Erhalt der Mobilität sowie die Prävention von Stürzen
		Die EASO-Position [40] empfiehlt bei älteren Menschen mit Adipositas ein multikomponentiges Training aus Flexibilität, Balance, aerobem Training (150–200 min/Woche moderat) und Krafttraining, individualisiert nach dem FITT-Prinzip (Frequency, Intensity, Time, Type); therapeutische Ziele sind Muskelerhalt, Mobilität, Selbstständigkeit und Lebensqualität [1]	

*EASO* European Association for the Study of Obesity, *NICE* National Institute for Health and Care Excellence, *W*HO World Health Organization

Bei Erwachsenen mit Übergewicht oder Adipositas scheinen kombinierte Ausdauer- und Krafttrainingsprogramme besonders günstig für kardiometabolische Endpunkte zu sein [40, 41]. Neben der Gewichtsreduktion ist der Erhalt von Muskelmasse, Kraft und körperlicher Funktion zentral, insbesondere im Kontext einer inkretinbasierten Therapie [42]. Auch bei Kindern und Jugendlichen sollten neben BMI und Körperfett vor allem Fitness, motorische Kompetenz, Selbstwirksamkeit und psychosoziale Teilhabe berücksichtigt werden; regelmäßiges aerobes Training über mindestens 12 Wochen zeigt hier positive Effekte auf Gewicht, BMI, Taillenumfang und Körperfett [43, 44].

## Diskussion

Körperliche Aktivität spielt in der Prävention und Therapie der Adipositas aus vielerlei Hinsicht eine bedeutsame Rolle. Aus Public-Health-Perspektive ist entscheidend, dass Bewegungsförderung nicht allein als individuelle Verhaltensanforderung verstanden wird. Adipositas entsteht in Lebenswelten, die durch energiedichte Ernährung, motorisierte Mobilität, sitzende Arbeit, Bildschirmzeiten, unsichere Bewegungsräume und soziale Ungleichheit geprägt sind [45]. Der WHO Global Action Plan on Physical Activity 2018–2030 fordert einen gesamtgesellschaftlichen Ansatz, der körperliche Aktivität über sichere aktive Mobilität, bewegungsfreundliche Orte, inklusive Programme und politische Rahmenbedingungen über die gesamte Lebensspanne fördert [45]. Auch die deutsche S3-Leitlinie zur Prävention der Adipositas empfiehlt auf Bevölkerungsebene ausdrücklich evidenzinformierte Maßnahmen zur Schaffung bewegungsfördernder Verhältnisse, weist zugleich aber darauf hin, dass die Wirksamkeit solcher Interventionen kontext- und umsetzungsabhängig ist und die Evidenzstärke variiert [6, 7].

Integriert werden sollte explizit die Reduktion vermeidbarer Sitzzeiten und die Förderung von Alltagsaktivität als besonders niedrigschwellige Ansatzpunkte. Sie erreichen auch Personen, für die strukturierter Sport aus körperlichen, zeitlichen, psychosozialen oder finanziellen Gründen schwer zugänglich ist. Auch alltagsnahe Strategien wie Gehen, Treppensteigen, aktive Wege, Bewegungspausen, Exercise Snacks oder kurze Kraftübungen können eine wichtige Brücke zu mehr Aktivität bilden. Eine konzeptionelle Ergänzung für die Public-Health-orientierte Bewegungsförderung kann Physical Literacy darstellen. Darunter wird das Zusammenspiel aus Motivation, Selbstvertrauen, körperlicher Kompetenz, Wissen und Verständnis verstanden, das Menschen befähigt, körperliche Aktivität als bedeutsam zu erleben und langfristig in ihren Alltag zu integrieren [46]. Der Mehrwert des Konzepts liegt darin, dass es Bewegungsförderung nicht auf Information oder individuelles Verhalten reduziert. Damit kann Bewegung nicht als kurzfristige Maßnahme zur Gewichtsreduktion, sondern als lebenslange Gesundheitsressource verstanden werden [47].

Einschränkend ist zu berücksichtigen, dass es sich bei diesem Artikel um eine narrative Übersicht handelt und keine eigene systematische Recherche mit formaler Bewertung des Verzerrungsrisikos durchgeführt wurde. Außerdem ist die aktuelle Evidenz insbesondere dort heterogen, wo konkrete adipositasbezogene Endpunkte und spezifische Bewegungsformate beurteilt werden. Dazu zählen Studiendesign, Populationen, Messverfahren, Interventionsart, Dosis und Nachbeobachtungsdauer [28–30, 35–38], was bei der Bewertung der jeweiligen Effekte und der Übertragbarkeit einzelner Interventionsformate in Alltag und Versorgung berücksichtigt werden muss. Für niedrigschwellige Ansätze wie Exercise Snacks liefern die bisherigen Studien wertvolle Hinweise zu Machbarkeit, Adhärenz und Fitness, jedoch sind Langzeitdaten zu Körpergewicht, Körperzusammensetzung und Nachhaltigkeit weiterhin begrenzt [37, 38]. Künftig sind daher pragmatische, längerfristig angelegte Studien unter Alltagsbedingungen wünschenswert, die neben Gewicht auch Körperzusammensetzung, Fitness, Teilhabe, Adhärenz und strukturelle Rahmenbedingungen berücksichtigen.

Zusammengefasst können aber individuelle, motivierende Beratungen, wie beispielsweise über das Rezept für Bewegung, der Einsatz digitaler Tools, Wearables oder Schrittziele hilfreich sein, bleiben jedoch begrenzt, wenn sichere Wege, bezahlbare Angebote, inklusive Sporträume, bewegungsfreundliche Schulen, arbeitsplatzbezogene Bewegungsoptionen und stigmavermeidende Gesundheitsversorgung fehlen [45]. Bewegungsförderung in der Adipositasprävention stellt daher zugleich eine medizinische, pädagogische, kommunale und gesundheitspolitische Aufgabe dar.

## Fazit

Körperliche Aktivität ist eine zentrale Säule der Adipositasprävention und -therapie über die gesamte Lebensspanne. Auch wenn Bewegung allein bei Erwachsenen meist nur eine moderate Gewichtsabnahme bewirkt, sind die positiven Effekte auf Körperzusammensetzung, metabolische Gesundheit, Fitness und Lebensqualität bedeutsam. Neben alters- und bedarfsgerechten Bewegungsangeboten bieten Alltagsaktivität und die Reduktion vermeidbarer Sitzzeiten niedrigschwellige Ansatzpunkte. Nachhaltige Bewegungsförderung erfordert, individuelle Befähigung mit bewegungsfreundlichen Lebenswelten, bezahlbaren und inklusiven Angeboten sowie einer stigmavermeidenden Versorgung zu verbinden. Physical Literacy kann diesen Ansatz ergänzen, indem Motivation, Selbstvertrauen, körperliche Kompetenz, Wissen und Verständnis gemeinsam in den Blick genommen werden. Forschungsbedarf besteht vor allem hinsichtlich längerfristiger Studien unter Alltagsbedingungen, die neben Gewicht und Körperzusammensetzung auch Fitness, Teilhabe, Adhärenz und strukturelle Rahmenbedingungen berücksichtigen. Nichtsdestotrotz sollte Bewegung als lebenslange Gesundheitsressource betrachtet und Bewegungsförderung als eine gemeinsame Aufgabe von Medizin, Bildung, Kommunen und Gesundheitspolitik vorangetrieben werden.

## Data Availability

Alle dieser Arbeit zugrunde liegenden Daten sind in diesem Artikel enthalten.
